# Cuproptosis: New Mechanisms Linking Endothelial Dysfunction to Neurodegenerative Diseases and Therapeutic Prospects

**DOI:** 10.3390/ijms27198765

**Published:** 2026-09-30

**Authors:** Tianyi Gu, Xiaoyu Wei, Shengyu Hua

**Affiliations:** College of Traditional Chinese Medicine, Tianjin University of Traditional Chinese Medicine, Tianjin 301617, China; gty19932870124@163.com (T.G.); tcmwxy@163.com (X.W.)

**Keywords:** cuproptosis, copper homeostasis, endothelial dysfunction, neurodegenerative diseases, mitochondrial metabolism, iron-sulfur cluster proteins

## Abstract

Cuproptosis is a copper-dependent form of regulated cell death driven by disrupted copper homeostasis and mitochondrial metabolic reprogramming. It is distinct from other cell death pathways and has been increasingly linked to vascular endothelial dysfunction and neurodegenerative diseases such as Alzheimer’s and Parkinson’s disease. This article comprehensively reviews the core molecular mechanisms of cuproptosis and its emerging roles in endothelial dysfunction and neurodegeneration, synthesizing recent findings from cellular, animal, and human studies. Cuproptosis exacerbates vascular pathology by impairing endothelial barrier function, inducing oxidative stress, and promoting inflammation and senescence. In the brain, it drives neurodegeneration both directly—via mitochondrial damage and neuronal loss—and indirectly by interacting with pathological proteins such as amyloid-beta and α-synuclein, creating a vicious cycle of copper dysregulation and protein aggregation. This review provides new insights into the pathophysiology of vascular endothelial dysfunction and neurodegenerative diseases by highlighting the pivotal role of cuproptosis. Understanding how cuproptosis links copper metabolism to cell death pathways opens avenues for identifying novel therapeutic targets. Interventions that restore copper homeostasis or block cuproptosis-specific molecular events may offer promising strategies for treating these devastating diseases.

## 1. Introduction

Cuproptosis is a novel form of regulated cell death first clearly defined in 2022. It is characterized by a reliance on intracellular copper accumulation, which triggers protein toxic stress and cell death by directly binding to and causing the oligomerization of lipoylated TCA cycle proteins [1,2]. This discovery has opened up a new field of research linking metal ion homeostasis to cell fate [3].

Cuproptosis differs mechanistically from known forms of cell death, such as apoptosis, necrosis, ferroptosis, and pyroptosis. Its core mechanism lies in the regulation of mitochondrial respiration, involving the binding of copper to acylated proteins in the citric acid cycle, leading to protein aggregation and the loss of iron–sulfur cluster proteins [2,3]. Key regulators of this process include ferredoxin 1 (FDX1), which induces cell death by reducing Cu^2+^ to Cu^+^, thereby promoting the acylation of dihydrolipoamide acetyltransferase (DLAT) and the degradation of iron–sulfur cluster proteins [3].

Endothelial cells serve as key regulators of vascular function, and their dysfunction lies at the core of cardiovascular and cerebrovascular diseases. Recent evidence suggests that copper homeostasis disruption is closely associated with endothelial injury. For example, the copper uptake transporter CTR1 in endothelial cells can sense disturbed blood flow and, by interacting with the mitochondrial copper transporter SLC25A3, which is primarily the mitochondrial phosphate carrier, has been suggested to participate in mitochondrial copper import, although its role remains debated and the identity of the authentic mitochondrial copper import pathway is still unresolved. According to the operational definition of cuproptosis proposed by Tsvetkov et al. [2], this elevated intramitochondrial copper may further enable FDX1 to reduce Cu^2+^ to Cu^+^, which directly binds to and induces the lipoylation-dependent oligomerization of dihydrolipoamide S-acetyltransferase (DLAT), accompanied by the loss of iron–sulfur cluster proteins (e.g., SDHB) and the induction of HSP70, ultimately triggering cuproptosis and exacerbating atherosclerosis. Notably, while numerous previous studies on endothelial cells have described general mitochondrial dysfunction induced by copper, we strictly classify those effects as broad ‘copper toxicity’ unless they specifically demonstrate the FDX1/lipoylation/Fe-S axis; our study focuses specifically on this defined cuproptotic pathway [4]. This finding reveals that the endothelial CTR1-SLC25A3-mitochondrial copper axis is a potential therapeutic target for atherosclerosis.

Furthermore, in conditions such as diabetes and hypertension—which serve as pathological precursors to cardiovascular and cerebrovascular diseases—endothelial cell death involves multiple forms of programmed cell death; cuproptosis, as a recently identified mechanism, is also thought to play a role in this process [5,6]. Furthermore, the brain is an organ with active copper metabolism, and disturbances in copper homeostasis have long been associated with the pathological processes of neurodegenerative diseases such as Alzheimer’s disease and Parkinson’s disease, although the specific mechanisms remain unclear [7]. Recent evidence suggests that cuproptosis may constitute a common pathway linking copper toxicity, endothelial injury, and neuronal loss [8,9,10]. Collectively, these studies suggest that copper homeostasis imbalance and the resulting cuproptosis may represent a key pathophysiological link between vascular endothelial dysfunction and neuronal degeneration in the central nervous system [7,11]. Despite these important advances, the current literature has predominantly investigated cuproptosis in isolation within neuronal or cancerous cell models, largely overlooking the indispensable role of the cerebrovascular endothelium.

Brain health and cognitive function are inherently dependent on the functional integrity of the vascular–neural unit. In this context, the endothelium serves a dual sentinel function: it acts as the first line of defense against circulating copper fluctuations and copper-bound lipoproteins, while simultaneously functioning as a potent inflammatory signal amplifier. Upon cuproptotic stress, endothelial cells not only undergo intrinsic mitochondrial collapse and energy failure but also release a cascade of damage-associated molecular patterns (DAMPs) and senescence-associated secretory phenotype (SASP) factors. This pathogenic cascade directly dismantles blood–brain barrier (BBB) integrity, flooding the neural parenchyma with toxic copper ions and pro-inflammatory cytokines (e.g., *IL-6*, *TNF-α*). Consequently, this creates a permissive “toxic soil” in the brain microenvironment, lowering the threshold for neuronal vulnerability and accelerating the aggregation of pathological proteins. Recognizing this vascular-to-neural continuum is essential to fully decipher the complex pathophysiology linking copper toxicity to neurodegeneration. While cuproptosis has been extensively studied in cancer and neuronal cell lines, its role in the vascular–neural interface remains poorly defined. Here we propose that endothelial cuproptosis serves as an upstream trigger that reshapes the neural microenvironment, lowering the threshold for proteinopathy and neuronal death.

To address this gap, this review is structured around five key domains: (i) the core molecular machinery of cuproptosis; (ii) endothelial vulnerability and barrier disruption; (iii) the role of endothelial damage in remodeling the neural microenvironment; (iv) reciprocal synergy between cuproptosis and Aβ/α-synuclein pathologies; and (v) therapeutic strategies and translational challenges. Each of these interconnected pillars is discussed in detail below.

## 2. The Core Molecular Mechanisms and Regulatory Networks of Cuproptosis

### 2.1. Transport, Chelation, and Intracellular Accumulation of Copper Ions

The intracellular homeostasis of copper ions is critical for maintaining normal physiological functions, and any disruption of this balance serves as a prerequisite for triggering pathological processes such as cuproptosis. Cu(I) ions primarily enter cells via the high-affinity copper transporter 1 (SLC31A1/CTR1) [12]. In the nervous system, CTR1 is expressed in various cell types, and its expression levels are strictly regulated [13,14]. Once inside the cytoplasm, copper ions are transported to specific subcellular targets by metal chaperones such as antioxidant 1 copper chaperone (ATOX1) and copper chaperone for superoxide dismutase (CCS), or bound to metallothioneins (MTs) for storage in an inert form, thereby preventing the toxicity of free copper ions [15].

Abnormally elevated levels of free copper ions within cells are a prerequisite for cuproptosis, which typically results from dysfunction of the copper efflux system or excessive copper intake due to environmental or genetic factors. Copper efflux is primarily mediated by the P-type ATPase transporters ATP7A and ATP7B [15]. In Wilson’s disease (WD), pathogenic mutations in the *ATP7B* gene impair the excretion of copper into bile, leading to pathological accumulation of copper in tissues such as the liver and central nervous system [16]; dysfunction of ATP7A, on the other hand, is associated with Menkes disease, leading to copper deficiency [17]. In pathological conditions such as cancer, upregulation of ATP7A and ATP7B expression is closely linked to resistance to platinum-based drugs, as these proteins facilitate the efflux of such drugs [18,19]. Studies have shown that excessive copper supplementation (e.g., up to 200% of the recommended intake) significantly increases serum and liver copper levels in middle-aged rats, alters vascular contractility, and induces oxidative stress [20].

Copper ions can enter mitochondria via specific transporters [21]. Recent studies have shown that dysregulation of copper uptake mechanisms in specific organelles, particularly mitochondria, leads to mitochondrial copper pool overload, which is a key event in the initiation of cuproptosis. As the central site for energy metabolism and the execution of cuproptosis, mitochondrial copper homeostasis is of critical importance; furthermore, copper ion carriers or copper complexes can efficiently deliver copper to mitochondria, inducing mitochondrial dysfunction and cuproptosis [22].

Excess copper in mitochondria binds to lipoylated proteins of the citric acid cycle, leading to abnormal oligomerization of these proteins, disrupting mitochondrial metabolism, and ultimately resulting in cell death [23]. In a pulmonary fibrosis model, copper ion transporter-induced bronchial epithelial cell death was accompanied by elevated intracellular Cu^+^ levels and DLAT oligomerization; this process could be reversed by the copper chelator tetrathiomolybdate (TTM), confirming the central role of mitochondrial copper overload [24].

Beyond the cytoplasmic chaperones described above, eukaryotic cells rely on a set of additional copper chaperones dedicated to mitochondrial copper delivery, which is essential for cytochrome c oxidase (CCO/COX) assembly. Copper transport into mitochondria is mainly mediated by the metallochaperone COX17 (cytochrome c oxidase copper chaperone 17), which carries Cu(I) from the cytoplasm to the mitochondrial intermembrane space (IMS) and inner-membrane proteins [25]. Within the IMS, COX17 functions as a central and specific Cu(I) donor to two distinct targets, the inner-membrane proteins SCO1 and COX11 [16,26]. COX11 and SCO1 serve as copper donors for the CuB and CuA copper sites of CCO, respectively, while SCO1 and SCO2 cooperate to deliver copper into the COX2 subunit [25,26]. The biogenesis of COX2 further requires COA6, a thiol–disulfide oxidoreductase structurally tuned to work together with SCO1/SCO2 during copper delivery to COX2 [27,28]; accordingly, COA6 deficiency impairs COX2 biogenesis and can be partially rescued by copper supplementation [29]. In parallel, COX19 and COX23—both twin-*CX9C*-motif proteins sharing homology with COX17—participate in this pathway: COX19 binds Cu(I), is required for COX11 function [29,30], and also transduces a SCO1-mediated mitochondrial redox signal that regulates *ATP7A*-dependent cellular copper efflux [31], whereas COX23 functions together with COX17 in a common copper-assembly pathway, acting upstream of COX17 [32]. In addition to these mitochondrial chaperones, the oncogenic protein MEMO1 (mediator of cell motility 1) coordinates reduced, but not oxidized, copper ions and interacts with ATOX1, thereby sequestering Cu(I) and protecting cells against copper-mediated reactive oxygen species (ROS) generation [33]. Collectively, this network of copper chaperones ensures precise, compartmentalized copper metallation of respiratory-chain enzymes; its dysregulation perturbs mitochondrial copper homeostasis, which lies at the interface of cuproptosis susceptibility.

Consequently, targeting mitochondrial copper transport or chelating excess copper within mitochondria has become a key strategy for intervening in cuproptosis-related diseases.

### 2.2. The Mitochondrial Respiratory Chain and the Oligomerization of Lipoylated TCA Cycle Proteins

Cuproptosis is characterized by the direct targeting and disruption of mitochondrial metabolic function by excess copper ions [34].

Specifically, copper ions bind specifically to acylated enzymes in the mitochondrial citric acid cycle, with dihydrolipoamide S-acetyltransferase being a key target [35]. This binding is not random but depends on the FDX1-mediated acylation process, which reduces copper ions to the more reactive Cu^+^, enabling them to bind stably to acylation sites [3]. The FDX1-mediated process, in which FDX1 serves a dual role: it reduces Cu(II) to Cu(I) and is also required for protein lipoylation via its interaction with lipoic acid synthase (LIAS), thereby enabling copper to bind to lipoylated sites on TCA cycle enzymes. Once bound, the copper ions induce abnormal oligomerization of these lipoylated TCA cycle proteins, forming insoluble protein aggregates [36]. This oligomerization process directly leads to the loss of function of the relevant enzymes, severely disrupting the normal operation of the TCA cycle and consequently triggering a complete collapse of mitochondrial metabolism, manifested as impaired oxidative phosphorylation, reduced ATP synthesis, and decreased activity of mitochondrial respiratory chain complexes [37].

It is worth noting that this cell death pathway exhibits unique biochemical characteristics; it does not rely on classical apoptosis, necroptosis, or ferroptosis signaling pathways [38]—its hallmark events include the aggregation of lipoylated proteins, the loss of iron–sulfur cluster proteins, and the upregulation of heat shock protein 70—and these features collectively constitute the molecular fingerprint that distinguishes cuproptosis from other forms of cell death [39].

Therefore, the targeting of lipoylated TCA cycle proteins in the mitochondrial respiratory chain by copper ions and the induction of their oligomerization represent the central biochemical events driving this unique cell death program.

### 2.3. Inhibition of Iron–Sulfur Cluster Biosynthesis and the Energy Crisis

However, one of the key mechanisms by which copper toxicity disrupts cellular energy homeostasis lies in its direct impairment of iron–sulfur cluster biosynthesis.

Iron–sulfur clusters serve as cofactors for numerous key proteins; they are widely distributed in the cytoplasm and mitochondria and are essential for maintaining cellular metabolism, particularly the function of the mitochondrial electron transport chain. Studies have shown that excess copper ions, particularly their reduced form Cu^+^, can directly target and inhibit the biosynthetic machinery of iron–sulfur clusters. This inhibitory effect has been confirmed in mice and cells used as models of Wilson’s disease (a disorder characterized by copper homeostasis imbalance). Copper overload inhibits the assembly of Fe-S clusters, reduces the activity of Fe-S enzymes, and leads to mitochondrial dysfunction [40]; furthermore, Cu^+^ generated by the copper-reducing activity of histone H3 has been shown to be a major disruptor of the functional pool of Fe-S clusters; in a yeast model of Friedreich’s ataxia, reducing Cu^+^ levels restores Fe-S cluster-dependent metabolism and growth [41]. Together, these findings reveal that copper toxicity sets the stage for subsequent mitochondrial dysfunction by interfering with the early steps of iron–sulfur cluster biosynthesis (Figure 1).

Widespread loss of function in iron–sulfur cluster proteins directly lead to severe mitochondrial dysfunction and a cellular energy crisis. Complexes I, II, and III of the mitochondrial electron transport chain all contain iron–sulfur clusters, which are the core components responsible for catalyzing electron transfer. When copper toxicity causes these iron–sulfur cluster proteins to become unstable or lose function, the integrity of the electron transport chain is disrupted, oxidative phosphorylation is impaired, and cellular ATP production drops sharply [42,43]—this energy crisis, acting in concert with the disruption of the citric acid cycle, pushes the cell to the brink of death.

During cuproptosis, copper ions bind directly to acylated proteins in the tricarboxylic acid (TCA) cycle, leading to abnormal oligomerization of these proteins. This not only disrupts the metabolic flux of the TCA cycle but also triggers subsequent events—specifically, the loss of iron–sulfur clusters—which further exacerbate the collapse of mitochondrial respiratory function [42,44]. This protein-toxic stress, jointly triggered by protein aggregation and iron–sulfur cluster loss, is the hallmark that distinguishes cuproptosis from other forms of cell death [45,46]. Therefore, the inhibition of iron–sulfur cluster biosynthesis and the resulting inactivation of protein function serve as the critical link connecting copper overload, the collapse of mitochondrial energy metabolism, and ultimate cell death.

## 3. Mechanisms of Cuproptosis-Induced Endothelial Dysfunction

### 3.1. Characteristics of Copper Homeostasis and Susceptibility in Endothelial Cells

As the inner lining of blood vessel walls, endothelial cells are directly exposed to circulating blood, which poses unique challenges for maintaining their copper homeostasis.

Before detailing the endothelial consequences of copper dyshomeostasis, a critical semantic and mechanistic clarification is warranted. Canonical cuproptosis is strictly defined as an FDX1-dependent, lipoylation-dependent, and ROS-independent cell death pathway. However, a substantial portion of the endothelial literature cited herein describes copper-induced oxidative stress, mitochondrial ROS production, and inflammatory injury that are rescued by antioxidants—features that are incompatible with the ROS-independent definition of cuproptosis. Therefore, throughout this section, we explicitly distinguish true cuproptosis from the broader category of copper toxicity (or copper-induced endothelial dysfunction). Unless the original study demonstrates FDX1 involvement and lack of ROS dependency, we re-label such observations as copper toxicity, while acknowledging that endothelial cells remain a critical sentinel of copper dyshomeostasis.

Fluctuations in blood copper ion levels directly affect endothelial cells. Under pathological conditions such as inflammation and oxidative stress, endothelial cells exhibit increased copper uptake and/or reduced copper efflux, making them a cell type susceptible to cuproptosis [47]. For example, in Alzheimer’s disease models, Aβ42 treatment upregulates the expression of the copper uptake protein CTR1 in human cerebral microvascular endothelial cells while downregulating the copper efflux protein ATP7A, leading to intracellular copper accumulation and oxidative stress, which disrupts endothelial barrier function [48]. This represents a prototypical example of copper-induced oxidative injury rather than canonical cuproptosis, as the reported cell damage is rescued by ROS scavengers—a finding incompatible with the ROS-independent nature of cuproptosis.

Imbalances in endothelial cell copper homeostasis result not only from increased uptake but are also closely linked to their unique regulatory network of copper transporters. The copper transporter P-type ATPase ATP7A plays a critical role in endothelial cell function. Not only does it respond to vascular endothelial growth factor (VEGF) stimulation by translocating from the Golgi apparatus to the plasma membrane, but it also promotes angiogenesis by inhibiting the binding of the autophagy adaptor protein p62 to ubiquitinated VEGFR2, thereby limiting the autophagy–lysosomal degradation of VEGFR2 [49]; more importantly, the stability of ATP7A itself is regulated: Studies have shown that caveolin-1 can bind to ATP7A to prevent its ubiquitination and proteasomal degradation, thereby stabilizing *ATP7A* expression, ensuring the activity of extracellular superoxide dismutase, and protecting blood vessels from oxidative stress damage [50]. When this stabilizing mechanism is impaired, ATP7A levels decrease, leading to reduced antioxidant defense capacity and exacerbated endothelial dysfunction.

Therefore, due to their unique anatomical location and delicate, fragile copper regulatory mechanisms, endothelial cells are highly susceptible to becoming the primary targets of copper toxicity in various pathological conditions; disruption of copper homeostasis is a key link connecting endothelial dysfunction to subsequent diseases (such as atherosclerosis and neurodegenerative diseases).

### 3.2. Disruption of Endothelial Barrier Function Caused by Cuproptosis

As described above, the core mechanism of cuproptosis lies in the abnormal accumulation of copper ions within mitochondria, leading to the aggregation of lipoylated proteins and dysfunction of the mitochondrial respiratory chain, which ultimately results in cellular energy depletion [51]. In endothelial cells, this process directly impairs normal mitochondrial function, resulting in severe impairment of adenosine triphosphate (ATP) synthesis [52]. The energy crisis deprives the maintenance and reorganization of the cytoskeleton of its power source, particularly disrupting the stability of actin stress fibers.

This structural failure has been substantiated in multiple disease models. Studies in acute cerebral infarction models have shown that the upregulation of cuproptosis-promoting proteins such as SLC31A1 and FDX1 is closely associated with mitochondrial dysfunction. Inhibition of cuproptosis has been shown to alleviate blood–brain barrier damage, which indirectly confirms the critical role of copper-mediated mitochondrial dysfunction and energy depletion in the disruption of the endothelial barrier [53]. Similarly, in temporal lobe epilepsy (TLE), single-cell analysis links the cuproptosis-associated gene *PDE5A* to BBB permeability [51].

Copper overload in endothelial cells often triggers severe oxidative stress, as copper ions catalyze hydroxyl radical generation via Fenton-like reactions, and mitochondrial dysfunction amplifies ROS production [52]. However, this oxidative damage must be distinguished from canonical cuproptosis, which is defined as a ROS-independent process driven by FDX1-mediated lipoylated protein aggregation and iron–sulfur cluster loss. In this review, we ascribe the above oxidative events to copper-induced oxidative injury rather than to cuproptosis itself. This heightened oxidative stress inflicts devastating damage on the intercellular junctions of endothelial cells. Intercellular junctional proteins, such as vascular endothelial cadherin (VE-cadherin), are central molecules in maintaining the adhesive junctions of endothelial cells and are highly sensitive to oxidative damage. Oxidative stress can lead to the degradation, loss of function, or abnormal localization of these junctional proteins, thereby disrupting the integrity of tight junctions and focal adhesions [54]. This disruption of junctional structures causes the originally tight endothelial layer to become riddled with gaps, directly leading to the leakage of plasma components into the extravascular tissue.

The critical consequence of this structural disintegration—beyond the endothelial injury itself—is that it permits blood-derived toxicants, including plasma proteins (thrombin, plasmin), inflammatory mediators, and free copper ions, to extravasate into the perivascular space, thereby initiating a cascade of parenchymal damage [48,53]. For example, in atherosclerosis (AS), endothelial dysfunction serves as the initiating step of the disease [55]. Following the disruption of the endothelial barrier, lipids and inflammatory cells can more easily penetrate the vascular endothelium, driving plaque formation. Similarly, studies combining bioinformatics analysis with machine learning models have revealed a link between cell death and the disruption of vascular wall integrity [56]. Thus, cuproptosis disrupts the cytoskeleton and intercellular junctions by triggering a mitochondrial energy crisis and oxidative stress in endothelial cells, ultimately leading to the loss of endothelial barrier function—a common pathological basis for various vascular pathologies including atherosclerosis, aneurysms, and neurovascular diseases.

### 3.3. Induction of Inflammatory and Senescent Phenotypes in Endothelial Cells

The process of cuproptosis profoundly affects the homeostasis of endothelial cells and induces significant inflammatory responses and aging phenotypes, which actively propagate inflammatory signals into the brain through at least two routes (Figure 2).

When endothelial cells experience copper overload, the disruption of copper homeostasis leads to mitochondrial dysfunction and the accumulation of lethal ROS [57]. This state of copper toxicity is distinct from cuproptosis, which does not require ROS for its execution. Thus, the findings are interpreted here as evidence of copper-induced oxidative stress rather than canonical cuproptosis. This severe cellular stress prompts the release of damage-associated molecular patterns (DAMPs), endogenous danger signals that activate pattern recognition receptors on the endothelial cells themselves and on surrounding immune cells, thereby initiating and amplifying inflammatory cascades [54]. Consistently, exposure to copper-containing particulate matter induces a pro-inflammatory and pro-fibrotic response in endothelial cells (e.g., secretion of *TNF-α* and *ICAM-1*) via oxidative stress mechanisms [58]. This response reflects copper-induced inflammatory injury rather than the specific FDX1-dependent cuproptotic cascade and is therefore discussed here as a consequence of copper toxicity. This inflammatory microenvironment triggered by cuproptosis, through the sustained secretion of pro-inflammatory factors such as interleukin-1β (*IL-1β*) and *IL-6*, not only exacerbates the inflammatory state of the local vascular wall but may also affect distal organs via the circulatory system, providing a potential vasculogenic explanation for the chronic neuroinflammation observed in neurodegenerative diseases [59].

Mitochondrial damage triggered by copper accumulation is a central mechanism underlying the accelerated aging of endothelial cells [60]. In many reported models, this damage is attributed to ROS-mediated oxidative stress, which again underscores the gap between copper toxicity and true cuproptosis. Here, we classify these events as copper-induced mitochondrial dysfunction and senescence, reserving the term cuproptosis for the FDX1/lipoylation-dependent, ROS-independent pathway (Figure 2).

During the aging process, a significant increase in copper ion levels is closely associated with vascular aging, and cuproptosis serves as the key link between copper homeostasis imbalance and vascular aging [57]. Endothelial cells subjected to cuproptosis stress exhibit a typical senescence-associated secretory phenotype (SASP). SASP is characterized by the massive secretion of pro-inflammatory factors, chemokines, growth factors, and matrix metalloproteinases, thereby creating a chronic pro-inflammatory and pro-fibrotic microenvironment [61]. For example, in the aging heart, myocardial ischemia–reperfusion injury specifically induces copper death in RGCC-expressing capillary endothelial cells. This loss of cells further compromises the integrity of the microvascular network and exacerbates tissue fibrosis and functional decline through the release of SASP factors [62].

Beyond passive leakage of soluble factors, the damaged BBB permits direct extravasation of pro-inflammatory cytokines and DAMPs into the perivascular space, where they activate pattern recognition receptors (e.g., *TLRs*) on resident microglia and astrocytes [63]. More importantly, endothelial cells undergoing cuproptosis release a surge of cell-specific exosomes that encapsulate inflammatory mediators, miRNAs, and copper-laden proteins. These exosomes traverse the compromised barrier or act locally to transfer pathogenic cargo to glial cells [64]. Upon receiving these vascular-derived signals, microglia shift toward a pro-inflammatory M1 phenotype (releasing additional *IL-1β* and *TNF-α*), while astrocytes adopt a neurotoxic A1 phenotype, amplifying the inflammatory cascade [65,66]. This vascular-to-glial inflammatory relay establishes a self-perpetuating “firestorm” within the brain parenchyma, transforming an initially local endothelial event into a widespread driver of synaptic dysfunction and neuronal vulnerability. Thus, cuproptosis transforms acute cell death events into a persistent chronic inflammatory state by inducing SASP in endothelial cells. This microenvironment not only impairs vascular function but may also play a significant role in the onset and progression of neurodegenerative diseases by disrupting the blood–brain barrier and promoting neuroinflammation [67].

### 3.4. The “Copper Overflow” from Blood Vessels into Brain Parenchyma

In addition to inflammatory mediators, copper ions themselves represent a primary toxic agent released from the vascular compartment. Under physiological conditions, the brain endothelium tightly regulates copper flux via the CTR1/ATP7A balance [68,69,70]. However, under cuproptotic stress, this balance is pathologically shifted toward net intracellular accumulation. When these overloaded endothelial cells succumb to death and lose membrane integrity, the accumulated intracellular copper—along with copper bound to albumin and other carriers—is passively liberated into the perivascular space [71,72].

We propose the “copper overflow” hypothesis, which posits that the dying endothelium acts as a substantial source of pathological copper excess in the neural microenvironment. This spillover directly elevates the extracellular labile copper pool available to neighboring neurons. Importantly, this overflowed copper is highly bioavailable and readily interacts with pathological proteins: in AD, it binds Aβ monomers to promote oligomerization and fibril formation [73,74]; in Parkinson’s disease, it stabilizes β-sheet-rich α-synuclein oligomers [75,76,77,78]. Therefore, endothelial cuproptosis-driven copper overflow provides the very metal cofactors required for proteinopathy propagation. By lowering the local threshold for pathological aggregation, this copper-rich milieu synergizes with neuronal mitochondrial defects to accelerate neurodegeneration.

## 4. Exploring the Link Between Cuproptosis and Neurodegenerative Diseases

### 4.1. The Pathological Link Between Cuproptosis and Alzheimer’s Disease

#### 4.1.1. The Interplay Between Disrupted Copper Homeostasis in the Brain and Aβ Pathology

In the brains of patients with Alzheimer’s disease (AD), there is a significant abnormal accumulation of copper in specific regions, such as around amyloid plaques [79]; this abnormal distribution of copper is closely associated with the pathological progression of AD.

Copper is a key trace element essential for maintaining normal brain function. As a cofactor for numerous important enzymes, it plays a role in energy metabolism, neurotransmitter synthesis, and synaptic transmission; the precise regulation of its homeostasis is critical for neuronal function [80]. However, in the brains of individuals with Alzheimer’s disease (AD), copper homeostasis is disrupted, manifesting as abnormally elevated local copper concentrations, particularly in regions rich in Aβ plaques [26]. Studies have shown that these free or abnormally bound copper ions can interact with Aβ peptides with high affinity, forming Cu-Aβ complexes [73]. This interaction has dual pathological effects: on the one hand, copper ions can catalyze conformational changes in Aβ monomers, accelerating their aggregation into oligomers and fibrils, thereby promoting the formation of amyloid plaques [74]; on the other hand, the Cu-Aβ complex itself possesses redox activity, capable of catalyzing molecular oxygen to generate ROS, such as hydrogen peroxide, triggering oxidative stress, which leads to neuronal lipid peroxidation, protein glycation, and DNA damage, thereby exacerbating neurotoxicity [81].

It is worth noting that Aβ oligomers themselves possess a strong ability to chelate copper, enabling them to capture copper ions from the environment and create a positive feedback loop: copper promotes Aβ aggregation, while aggregated Aβ further chelates more copper, leading to a vicious cycle of continuously rising local copper concentrations and oxidative damage [82].

Recent studies have further elucidated the complex role of copper-Aβ complexes in cell death pathways, particularly their potential link to cuproptosis. In the context of AD, disruptions in brain copper homeostasis—especially the abnormal accumulation of copper around Aβ plaques—may indirectly increase cellular susceptibility to cuproptosis by interfering with the normal distribution of copper in neurons and glial cells. For example, Aβ oligomers can upregulate the expression of the copper transporter SLC31A1 (CTR1) in neurons, thereby increasing cellular copper uptake [83]. Once intracellular copper ion levels exceed physiological thresholds—particularly when they accumulate in mitochondria—this may trigger the cuproptosis pathway [84]. Furthermore, glial cells, particularly astrocytes, play a crucial “copper sponge” role in regulating brain copper homeostasis, responsible for taking up copper from the blood to buffer its neurotoxicity and delivering it to neurons when needed [85]. However, under AD pathological conditions, Aβ deposition and associated inflammatory responses may impair the copper-buffering function of astrocytes [86]. This dysfunction of glial cells, coupled with an imbalance in the neurons’ own copper uptake and efflux, collectively creates a microenvironment prone to copper toxicity and cuproptosis.

Therefore, Aβ pathology not only damages neurons through direct oligomer toxicity and oxidative stress but may also accelerate neurodegenerative processes by disrupting the spatiotemporal distribution of copper in the brain, thereby making neurons and glial cells more susceptible to the lethal effects of cuproptosis [66]. Understanding this cross-talk mechanism is crucial for developing novel AD treatment strategies that simultaneously target Aβ aggregation and copper homeostasis disruption.

#### 4.1.2. Cuproptosis Directly Drives Neuronal and Synaptic Damage

In various neurological injuries and diseases, cuproptosis has been shown to play a direct role in neuronal death and synaptic dysfunction, constituting a central pathological mechanism in neurodegeneration [11].

In models of acute central nervous system injury, such as following traumatic brain injury (TBI), cuproptosis is induced in both human patients and mouse models, peaking on day 3 post-injury and occurring primarily in neurons [87]. Following intracerebral hemorrhage (ICH), elevated copper levels and FDX1 expression, low expression of acylated DLAT and LIAS, loss of mitochondrial membrane potential, and alterations in neuronal damage markers collectively contribute to the eventual neuronal death [88]. These studies clearly demonstrate that the cuproptosis pathway plays a direct driving role in neuronal loss following acute injury.

The mechanism by which cuproptosis damages neurons profoundly affects synaptic integrity and function (Figure 3). Since mitochondrial dysfunction lies at the core of cuproptosis, the resulting mitochondrial collapse directly leads to energy (ATP) depletion in presynaptic terminals and impaired neurotransmitter release [11]. Studies have confirmed that copper exposure can lead to cognitive dysfunction in mice; the mechanism involves impairing synaptic plasticity by inhibiting the expression of PSD-95 and SYP, thereby affecting presynaptic and postsynaptic regulatory mechanisms [89]. In radiation-induced brain injury (RBI), spatial transcriptomics analysis has revealed the critical role of neuronal cuproptosis, and inhibiting this process effectively improves behavioral function and increases neuronal survival [90]; furthermore, in a subarachnoid hemorrhage (SAH) model, neuron-targeted anti-cuproptosis therapy alleviates oxidative stress, exerts neuroprotective effects, and improves neurobehavioral deficits [91].

Taken together, this evidence suggests that cuproptosis not only directly causes neuronal death but also indirectly leads to the loss of synaptic connections and dysfunction of neural networks by disrupting mitochondrial energy metabolism and protein homeostasis, which are essential for maintaining synaptic function.

In addition, certain genetic or pathological factors may render specific populations of neurons more susceptible to cuproptosis. For example, the apolipoprotein E ε4 (*ApoE ε4*) allele is the strongest genetic risk factor for Alzheimer’s disease (AD); neurons carrying this allele may exhibit mitochondrial metabolic defects, making them more vulnerable to copper ion accumulation and cuproptosis [68]. In the pathological context of AD, copper homeostasis is disrupted, and elevated levels of free copper, in particular, exacerbate oxidative damage and neurodegeneration. New evidence suggests that amyloid-β (Aβ) oligomerization promotes copper accumulation within neurons, wherein elevated Cu^+^ drives ROS production, lipoylated protein aggregation, and FDX1-dependent cuproptosis, while Cu^+^ chelation or *FDX1* knockdown can completely prevent cell death [92]. This suggests that in AD, Aβ pathology and the cuproptosis pathway may act synergistically to accelerate neuronal dysfunction. Similarly, in Parkinson’s disease (PD), the mitochondrial function of dopaminergic neurons is inherently fragile, and copper homeostasis disruption may exacerbate their degeneration by inducing cuproptosis [9]. In diabetes-associated cognitive dysfunction (DACD), exposure to high glucose and palmitic acid elevates intracellular copper levels and reduces the expression of FDX1, LIAS, and DLAT, thereby inducing cuproptosis; conversely, exogenous mitochondrial transplantation can improve cognitive deficits by inhibiting this process [93].

These findings highlight that, in various disease contexts, inherent metabolic vulnerabilities or proteinopathies may intersect with cuproptosis, jointly determining the fate of specific neuronal populations and accelerating their functional decline and degenerative processes.

#### 4.1.3. Cuproptosis-Specific Executors in Alzheimer’s Disease Pathology

Beyond the indirect effects mediated by copper-Aβ interactions, recent evidence has identified the direct engagement of cuproptosis core executors—particularly FDX1, LIAS, and DLAT—in AD pathogenesis. FDX1, the upstream reductase that converts Cu^2+^ to Cu^+^ and initiates the lipoylation-dependent cell death cascade, is significantly upregulated in the peripheral blood and neuronal models of AD patients, with its expression levels positively correlating with disease severity and cognitive decline [94]. This upregulation is not merely an epiphenomenon; in AD-affected neurons, elevated FDX1 activity drives the aberrant oligomerization of DLAT, which accumulates as insoluble aggregates in mitochondria, directly mirroring the hallmark biochemical lesion of cuproptosis [8,95]. LIAS, the enzyme responsible for lipoylation of TCA cycle proteins, also shows altered expression in AD brains, suggesting that the entire lipoylation-death axis is pathologically activated [70].

Critically, this molecular cascade is not independent of Aβ pathology but intimately intertwined. Aβ oligomers, through yet incompletely understood signaling pathways, can further upregulate FDX1 expression, creating a feed-forward loop: Aβ promotes FDX1-mediated cuproptosis, and the ensuing mitochondrial damage exacerbates oxidative stress, which in turn increases Aβ production and aggregation [92]. This synergy has been directly demonstrated in neuronal cultures, where Cu^+^ chelation or FDX1 knockdown completely prevented Aβ-induced cell death, positioning the FDX1-DLAT axis as a non-redundant downstream effector of Aβ neurotoxicity. Therefore, in AD, Aβ pathology and the cuproptosis pathway do not merely co-exist; they engage in a reciprocal amplification cycle that accelerates neuronal dysfunction beyond what either insult could achieve alone. This mechanistic insight suggests that targeting FDX1 or stabilizing lipoylated proteins may offer a novel therapeutic strategy distinct from conventional anti-Aβ immunotherapies.

#### 4.1.4. How Endothelial Cuproptosis Fuels AD Pathology: A Positive-Feedback Loop Between Copper Leakage and Inflammation

Under cuproptosis stress, endothelial cells not only lose their barrier integrity but also release large amounts of inflammatory mediators (*IL-6*, *TNF-α*) and damage-associated molecular patterns (DAMPs) through the SASP [96,97]. However, these vascular changes are not isolated events; rather, they constitute a “catalytic” microenvironment that exacerbates the core pathologies of AD—Aβ deposition and neuronal cuproptosis. Specifically, endothelial cuproptosis synergistically drives AD progression through two intersecting pathways: “copper spillover” and “inflammatory reprogramming.”

First, copper efflux directly provides the pathological basis for Aβ aggregation and lowers the threshold for copper-induced neuronal death. When the membrane integrity of endothelial cells undergoing cuproptosis is compromised, intracellular copper ions—particularly redox-active Cu^+^—are released in large quantities into the interstitial spaces of the brain parenchyma, causing a dramatic expansion of the “available copper pool” in the local microenvironment. According to the classic metal ion hypothesis, elevated concentrations of extracellular free copper ions catalyze the conformational transition of Aβ monomers through high-affinity binding, accelerating the formation of β-sheet structures and oligomerization, ultimately stabilizing amyloid plaques [98]. More critically, this copper-rich microenvironment significantly upregulates the expression of the copper-transport protein CTR1 (SLC31A1) on neuronal synaptic membranes [42]. The study found that under AD pathological conditions, Aβ42 oligomers themselves can directly upregulate SLC31A1 expression in cardiomyocytes via an unknown signaling pathway, accompanied by downregulation of the copper efflux protein ATP7A [83,99]. This leads to passive uptake of excessive copper ions by neurons in response to the extracellular “copper surge.” When mitochondrial copper loading exceeds the detoxification threshold mediated by FDX1, the neuron’s intrinsic cuproptosis pathway is abnormally activated [95,100,101]. Therefore, copper leakage from endothelial cells not only directly promotes Aβ deposition but also, by upregulating neuronal CTR1, establishes a direct pathological pathway: “from elevated extracellular copper to increased copper uptake by neurons, followed by the activation of mitochondrial cuproptosis, and ultimately leading to neuronal damage.”

Endothelial SASP inflammatory factors indirectly weaken the neuronal protective barrier by disrupting glial copper homeostasis. In the AD brain, astrocytes serve as critical “copper sponges,” responsible for buffering local excess copper ions and protecting neurons [102]. However, inflammatory factors such as *TNF-α* and *IL-1β*, released into the cerebrospinal fluid and interstitium following copper-induced endothelial injury, can act on neighboring astrocytes, inducing their transformation into the neurotoxic phenotype. This phenotypic shift is accompanied by a significant decline in astrocyte copper-buffering capacity and dysregulation of metallothionein (MT) expression, rendering them unable to effectively neutralize copper ions spilling over from the vascular side [103,104]. At the same time, the persistent inflammatory microenvironment activates M1-type polarization of microglia, leading to the release of more ROS and inflammatory mediators, which further reduces neurons’ ability to resist copper-induced proteotoxic stress [105].

It is evident that endothelial cuproptosis, neuroinflammation, and Aβ pathology form a self-perpetuating, destructive cycle through the mechanism of “copper efflux—upregulation of copper uptake—protein aggregation—re-injury of the endothelium.” This cycle significantly lowers the threshold for neurons to resist Aβ neurotoxicity, amplifying initial vascular damage into irreversible parenchymal neurodegenerative lesions.

### 4.2. The Potential Role of Cuproptosis in the Pathogenesis of Parkinson’s Disease

#### 4.2.1. Interactions Between α-Synuclein Aggregates and Copper Ions

Alpha-synuclein (α-synuclein, αSyn) is the primary constituent protein of Lewy bodies in neurodegenerative diseases such as Parkinson’s disease, and its abnormal aggregation is a central pathological feature of these conditions [106]. Studies have shown that αSyn has the ability to bind copper ions, and the regulatory effects of copper on αSyn aggregation are complex and concentration-dependent; it can bind both Cu(II) and Cu(I) with high affinity [107].

The N-terminal region of α-Syn contains multiple copper-binding sites, and N-terminal acetylation significantly alters its copper-binding properties [108]; molecular dynamics simulations further reveal that the binding of Cu(II) ions to α-Syn reduces the size and flexibility of the polypeptide chain, inducing more and more persistent secondary structural elements such as β-sheets and hairpin structures, thereby restricting its conformational space. This copper ion-mediated conformational change is considered a key step in promoting the transformation of α-Syn from soluble monomers to toxic oligomers and amyloid fibrils [109]. Molecular simulations indicate that, at different copper concentrations, α-Syn dimers form polymorphic structures through different copper-binding sites, which determines the molecular pathway of primary nucleation [110]; furthermore, copper ions can also interact synergistically with lipid membranes to regulate the kinetics and structure of α-Syn [111].

Excessively aggregated α-Syn may place dopaminergic neurons at risk of cuproptosis through various mechanisms. On the one hand, α-Syn’s high-affinity binding to copper ions may lead to the abnormal “hijacking” of the available copper pool within neurons, disrupting normal copper homeostasis. This disruption, in turn, may further trigger oxidative stress, mitochondrial dysfunction, and neuroinflammation—all of which are common pathways underlying cuproptosis and neurodegeneration [26]. On the other hand, the α-Syn-Cu complex itself may possess redox activity [112], which could catalyze the production of ROS, exacerbate oxidative damage, and impair mitochondrial function [26], with mitochondrial dysfunction being a central component of TCA cycle disruption and the execution of cuproptosis. Consequently, a vicious cycle forms between the abnormal aggregation of α-Syn and copper ion metabolism dysregulation: copper ions promote α-Syn aggregation and may enhance its toxicity, while aggregated α-Syn further disrupts copper homeostasis and mitochondrial function, ultimately potentially driving cells toward cuproptosis [9]. To address this interaction, the use of metal–protein attenuating compounds (MPACs) such as X1INH to competitively bind copper ions has been shown to attenuate α-Syn aggregation, providing a potential strategy for intervention [113].

#### 4.2.2. The Particular Vulnerability of Dopaminergic Neurons

Dopaminergic neurons exhibit particular vulnerability in neurodegenerative diseases, especially Parkinson’s disease, which is closely linked to their unique metabolic characteristics and inherent oxidative stress environment. These neurons are highly metabolically active, and the processes of dopamine synthesis and release themselves generate ROS, causing mitochondria to be subjected to sustained high levels of oxidative stress [114]. This inherent metabolic stress constitutes a fundamental vulnerability.

Furthermore, dopaminergic neurons are particularly sensitive to disturbances in copper homeostasis. As an essential redox-active biomineral, both the homeostasis and imbalance of copper are associated with pathological processes [115,116]. Chronic occupational or environmental exposure to copper has been shown to be associated with an increased risk of Parkinson’s disease and can induce the loss of dopaminergic neurons in the substantia nigra pars compacta [114,117]. In vitro and in vivo models have shown that copper exposure can directly cause dopaminergic neuron death through mechanisms involving the induction of oxidative stress, mitochondrial dysfunction, and the inhibition of the ubiquitin–proteasome system and autophagy [114]. This copper-mediated neurotoxicity is further amplified in the context of genetic susceptibility to Parkinson’s disease; for example, in models carrying *PARKIN* or *LRRK2* gene mutations, an excess supply of copper significantly exacerbates neurodegeneration, highlighting the synergistic role of genetic and environmental factors in dopaminergic neuron damage [118].

This inherent metabolic stress, combined with external copper homeostasis dysregulation, creates a cellular environment that allows cuproptosis to be triggered at a low threshold. As previously discussed, cuproptosis is a recently discovered form of programmed cell death that depends on mitochondrial function; its core mechanism involves the accumulation of copper ions within mitochondria, leading to the toxic aggregation of lipoylated proteins and oxidative damage; The high metabolic activity of dopaminergic neurons renders them highly sensitive to mitochondrial dysfunction. When compounded by Parkinson’s disease-associated defects in mitochondrial autophagy—such as the failure of mitochondrial quality control caused by abnormalities in the PINK1/Parkin pathway—damaged mitochondria cannot be cleared in a timely manner, and copper ions within them may accumulate more readily, triggering the cuproptosis pathway (Figure 4) [119]. Studies have confirmed that cuproptosis-related genes (CRGs) are consistently downregulated in samples from Parkinson’s disease patients, and these genes are significantly associated with immune cell infiltration and mitochondrial localization, suggesting a potential role for cuproptosis pathways in the immunometabolic dysregulation of Parkinson’s disease [120]. For example, changes in the expression of key cuproptosis genes such as FDX1 and LIAS may influence cell fate through the FDX1/LIAS signaling pathway [121].

Consequently, due to their high metabolic demands, inherent oxidative stress, and susceptibility to disturbances in copper homeostasis and defects in mitochondrial autophagy, dopaminergic neurons are more prone to the activation of the cuproptosis cell death mechanism, thereby accelerating their degenerative process in Parkinson’s disease. The vulnerability of dopaminergic neurons to cuproptosis is profoundly amplified by the PD-associated defects in mitochondrial quality control. Under physiological conditions, damaged mitochondria are cleared via the PINK1/Parkin-mediated mitophagy pathway [122,123]. However, in sporadic and familial PD, this clearance machinery is compromised—either by genetic mutations (*PINK1*, *Parkin*, *LRRK2*) or by acquired mitochondrial DNA damage. When mitophagy fails, aged and dysfunctional mitochondria accumulate within the perikarya and terminals of dopaminergic neurons [124,125,126,127,128]. These residual mitochondria are characterized by a decreased membrane potential and increased ROS production; most importantly, their ability to expel accumulated copper ions is likely impaired. As a result, the mitochondrial copper pool in these neurons reaches the threshold required for FDX1-dependent DLAT oligomerization much more readily than in cells with intact mitophagy. This provides a mechanistic explanation for the long-standing observation that environmental copper exposure preferentially damages dopaminergic neurons in genetically predisposed individuals [118]. It also suggests that enhancing mitophagy—for instance, through pharmacological activation of PINK1/Parkin—may indirectly raise the cuproptosis threshold and confer neuroprotection in PD, representing a conceptually novel combination strategy.

#### 4.2.3. Vascular Copper Spillover and Dopaminergic Vulnerability: The “Double-Hit” Effect of Mitochondrial Autophagy Defects

Regarding the pathological mechanisms of Parkinson’s disease (PD), the traditional view emphasizes the causal relationship between α-synuclein (α-Syn) aggregation and mitochondrial dysfunction. As discussed earlier, dopaminergic neurons possess an inherent vulnerability due to their high metabolic burden; this section further proposes that: The combination of copper release and defects in mitochondrial quality control constitutes a “double-hit” pathological model that induces cuproptosis. This provides a novel vascular pathological explanation for the interaction between environmental exposure and genetic susceptibility in sporadic PD.

Copper leakage from blood vessels provides a continuous, available source of copper for dopaminergic neurons [129]. However, copper leakage alone is not sufficient to specifically kill dopaminergic neurons; the key “second hit” lies in the fact that this population of neurons exhibits a significant defect in mitochondrial autophagy. Under physiological conditions, damaged mitochondria are specifically marked via the PINK1/Parkin pathway and transported to lysosomes for degradation; this is the core defense mechanism for clearing accumulated metal ions and oxidatively damaged proteins from mitochondria. However, in sporadic PD and familial PD associated with mutations in the *PINK1*, *Parkin*, or *LRRK2* genes, this mitochondrial quality control system is severely compromised [130,131,132,133]. When copper overflow causes a slight increase in copper ion concentration within the mitochondrial matrix, normal neurons may selectively clear “aged mitochondria” with excessive copper content by enhancing mitochondrial autophagy, thereby blocking the cuproptosis signal.

However, damaged mitochondria cannot be cleared in a timely manner. These residual mitochondria exhibit a decreased membrane potential, reduced respiratory chain complex activity, and severely impaired copper ion efflux capacity—as seen in the Wilson’s disease model—which relies on transporters such as ATP7B [134,135]. When copper ions from vascular leakage continuously flood these neurons—which are unable to clear waste—the copper ions accumulate in large quantities within the dysfunctional mitochondria, accelerating the onset of DLAT oligomerization [42]. Due to defects in mitochondrial autophagy, these mitochondria—which have accumulated copper ions and aggregated proteins—not only fail to be degraded but instead act as “apoptosis signal amplifiers”; accompanied by the accumulation of mitochondrial ROS and protein toxic stress, they ultimately accelerate the accumulation of copper load and dramatically amplify it into an irreversible copper-induced apoptotic signal [25,136].

In summary, we can say that vascular barrier dysfunction and peripheral copper leakage exploit vulnerabilities in the endogenous mitochondrial clearance mechanisms of dopaminergic neurons, thereby specifically lowering the threshold for neuronal cuproptosis. This insight broadens the focus of therapeutic interventions from simply clearing α-Syn or chelating systemic copper to comprehensive strategies aimed at restoring copper transport across the blood–brain barrier and enhancing neuronal mitochondrial autophagy reserves.

### 4.3. Cuproptosis and Other Neurodegenerative Diseases

#### 4.3.1. The Role of Cuproptosis in Huntington’s Disease and Mitochondrial Metabolic Defects

Huntington’s disease (HD) is an autosomal dominant neurodegenerative disorder caused by an abnormal expansion of the CAG trinucleotide repeat sequence in the huntingtin (*HTT*) gene [137]. One of the core pathological features of this disease is widespread and severe mitochondrial dysfunction and defects in energy metabolism, which are considered key factors contributing to the selective vulnerability of medium spiny neurons in the striatum [137,138].

Mutant HTT protein (mHTT) can directly target and damage mitochondria, triggering a series of functional abnormalities, including reduced activity of mitochondrial respiratory chain complexes, disruption of the tricarboxylic acid (TCA) cycle, excessive production of ROS, and mitochondrial dysregulation [139,140]. This underlying mitochondrial energy crisis renders neurons more vulnerable to additional stressors. Notably, copper homeostasis imbalance and its induced cuproptosis mechanisms may exert a synergistic amplifying effect with pre-existing mitochondrial metabolic defects in HD, thereby exacerbating neuronal death [141,142].

In the context of HD, the mitochondrial structural and functional damage caused by mHTT may render mitochondria more sensitive to copper-induced proteotoxic stress. For example, mHTT can induce increased mitochondrial DNA (mtDNA) heterogeneity and deletions, impairing electron transport chain function, which may thereby weaken the cell’s ability to process excess copper ions [143]; simultaneously, mitochondrial dysfunction itself may alter the intracellular reducing environment, affect the redox state of copper ions and further promoting the onset of cuproptosis [144].

Studies have also shown that mHTT expression alters the composition of mitochondrial membrane lipids, which may directly influence the aggregation behavior of the HTT protein and potentially disrupt the localization and function of copper-dependent enzymes within mitochondria, thereby creating a microenvironment conducive to copper ion-triggered cell death signals [145].

Therefore, the primary mitochondrial metabolic defects induced by mHTT in HD not only directly lead to insufficient energy supply to neurons and oxidative stress but may also significantly amplify the cuproptosis signaling pathways triggered by copper homeostasis imbalance, creating a vicious cycle that collectively drives disease progression [70,84]. Understanding this synergistic mechanism provides a crucial theoretical basis for developing novel combined therapeutic strategies that simultaneously target mitochondrial protection and copper homeostasis regulation.

#### 4.3.2. The Role of Cuproptosis in Amyotrophic Lateral Sclerosis

Amyotrophic lateral sclerosis (ALS) is a fatal, progressive neurodegenerative disease that primarily affects the upper and lower motor neurons in the brain and spinal cord, leading to muscle weakness, atrophy, and ultimately respiratory failure [146,147]. Although most cases of ALS are sporadic (SALS), approximately 10% are familial (FALS), with mutations in the superoxide dismutase 1 (*SOD1*) gene being one of the earliest identified and most extensively studied genetic factors. Recent studies have progressively revealed that imbalances in metal ion homeostasis, particularly copper homeostasis, and the newly defined copper-dependent cell death pathway—cuproptosis—play a key role in the pathogenesis of ALS [141,148,149,150].

Precise regulation of copper homeostasis is critical for maintaining neuronal function, and its disruption can directly lead to neurotoxicity. This disruption is particularly pronounced in ALS. SOD1, a major intracellular copper/zinc superoxide dismutase, relies heavily on the correct incorporation of the copper cofactor for its proper folding and enzymatic activity [84,150]. Extensive research has demonstrated that in SOD1-mutation-associated ALS (SOD1-ALS), the interaction between the mutant SOD1 protein and its copper chaperone, CCS (copper chaperone for *SOD1*), is impaired. This prevents the effective delivery of copper to the active site of SOD1, resulting in insufficient metallation and misfolding of SOD1. This incompletely metallated SOD1 is more prone to abnormal aggregation, forming cytotoxic aggregates, which is one of the core pathological features of ALS [151,152]. Furthermore, functional abnormalities in copper transporters also exacerbate copper homeostasis disruption. For example, the M1311V mutation in the *ATP7A* gene found in ALS patients leads to abnormal subcellular localization and impaired function of this protein, thereby hindering the compartmentalized transport of intracellular copper and causing abnormal copper accumulation [153].

Cuproptosis offers a new perspective on understanding the selective loss of motor neurons in ALS [26]. Recent studies using bioinformatics analysis have revealed that multiple genes associated with cuproptosis are differentially expressed in spinal cord or blood samples from ALS patients. For example, changes in the expression of LIAS, a key enzyme in the acylation pathway, may affect mitochondrial energy metabolism, while the expression levels of FDX1, an upstream regulator of cuproptosis, may be associated with the rate of disease progression [154]. These findings suggest that functional disruption of the cuproptosis regulatory network may represent an important pathway driving ALS neuronal degeneration, distinct from traditional apoptosis and necrotic apoptosis.

Abnormal copper accumulation can generate ROS via the Fenton reaction, exacerbating oxidative damage. In turn, the oxidative stress environment further promotes the misfolding and aggregation of proteins such as SOD1 and may impair the cell’s ability to buffer copper toxicity, for example, by depleting the key antioxidant glutathione (GSH) [155,156]. Metallothioneins (MTs), as important intracellular metal chelators and antioxidant proteins, are upregulated in ALS and are considered a compensatory protective mechanism. In ALS model mice, upregulation of MT1/2 or MT3 expression has been shown to mitigate oxidative damage, protect motor neurons, and prolong survival [157,158].

## 5. Prospects for Diagnostic and Therapeutic Strategies Targeting Cuproptosis

### 5.1. Development of Biomarkers

As a recently discovered copper-dependent form of programmed cell death, the development of specific biomarkers for cuproptosis is crucial for the early diagnosis, disease monitoring, and treatment evaluation of neurodegenerative diseases. An ideal biomarker should be able to sensitively and specifically reflect the activity of cuproptosis in vivo, particularly within the complex environment of the central nervous system [159,160]. Current research efforts are primarily focused on the development of molecular biomarkers in body fluids and brain-specific imaging probes.

With regard to biological markers, researchers are working to identify specific molecules in blood or cerebrospinal fluid that can indicate the process of cuproptosis. The core mechanism of cuproptosis involves the abnormal oligomerization of lipoylated proteins in the mitochondrial tricarboxylic acid cycle and the loss of iron–sulfur cluster proteins [7,142]; therefore, specific mitochondrial metabolic intermediates, fragments of oligomerized lipoylated proteins, or molecules reflecting protein toxic stress may serve as potential biomarkers [155,161]. For example, in studies of Alzheimer’s disease (AD), the key cuproptosis gene *FDX1* has been found to be significantly upregulated in patients’ peripheral blood and neuronal models, with its expression levels correlated with disease status, suggesting its potential as a diagnostic biomarker [8]; Furthermore, through bioinformatics analysis, researchers have identified multiple key genes associated with cuproptosis from the transcriptomic data of AD patients and constructed diagnostic models; changes in the expression profiles of these genes provide new insights for the development of peripheral blood-based diagnostic tools [162,163]. In addition to proteins and metabolites, non-coding RNAs play a crucial role in regulating the expression of genes associated with cuproptosis. Their stability in the peripheral circulation makes them a promising source of non-invasive biomarkers [164].

On the other hand, the development of molecular imaging probes capable of specifically detecting copper-mediated pathological processes in the brain is key to achieving in vivo, visual diagnostics. This is particularly relevant in cases where the blood–brain barrier prevents peripheral biomarkers from fully reflecting pathological changes in the central nervous system. PET tracers based on copper chelators represent a highly promising avenue. The design principle of these probes involves combining a chelator with high affinity and selectivity for copper ions with a radiotracer. When copper ions abnormally accumulate in the brain and trigger copper-mediated pathological processes, the probe specifically accumulates in the affected regions, thereby enabling visualization through imaging [42,165], similar strategies are being explored in other copper-related disorders [166]. In the future, the development of smart probes capable of distinguishing between copper ions of different valences or specifically binding to lipoylated proteins aggregated during cuproptosis will provide more precise pathological information [167].

Furthermore, by combining single-cell sequencing with spatial transcriptomics, it is possible to map the expression patterns of copper-related genes in different brain regions or cell types with greater precision, thereby guiding the targeted design of imaging biomarkers [38].

In summary, the integration of biofluid molecular biomarkers identified through multi-omics data analysis with advanced molecular imaging technologies will establish a multi-tiered, non-invasive or minimally invasive biomarker system, thereby significantly advancing the progress of precision medicine for cuproptosis-related neurodegenerative diseases.

### 5.2. Re-Evaluation and Targeted Application of Copper-Chelating Agents

Traditional copper chelators such as D-penicillamine and quinolamine have a long history of use in fields such as neurodegenerative diseases; however, their clinical efficacy has been inconsistent, prompting researchers to reevaluate their mechanisms of action and dosing strategies. For example, although D-penicillamine can bind copper ions, its systemic administration may deplete functional copper pools, leading to side effects, and it struggles to effectively cross the blood–brain barrier to reach the affected brain regions [168].

A study on doxorubicin-induced cardiomyopathy demonstrated that D-penicillamine exerts a cardioprotective effect by inhibiting FDX1-mediated cuproptosis. This suggests that its efficacy may depend heavily on precise intervention in specific pathological pathways (such as cuproptosis) rather than on non-selective systemic copper depletion [169]. Consequently, these new insights underscore the need to develop more precise delivery strategies, such as tailoring treatment regimens to specific disease stages or developing chelators capable of targeting specific organelles (e.g., mitochondria), in order to minimize off-target effects and ensure effective drug concentrations at the site of the lesion [170].

To overcome the limitations of traditional chelators, the development of novel copper chelators or modulators capable of crossing the blood–brain barrier and selectively clearing toxic copper pools has become a frontier in current research. The ideal strategy is not to distinguish between chemically identical copper ions, but rather to restore proper copper homeostasis—by redistributing ions to their correct subcellular compartments and/or supporting impaired cellular efflux mechanisms—thereby mitigating pathological copper accumulation while preserving essential cuproenzyme functions. Studies have shown that in AD models, Aβ42 upregulates the expression of the copper import transporter CTR1 and downregulates that of the export protein ATP7A, leading to copper accumulation in cerebral endothelial cells. This, in turn, disrupts the integrity of the blood–brain barrier through ROS-dependent mechanisms. In response, the use of membrane-permeable copper chelators or *CTR1* knockdown effectively inhibits this process [48], providing a theoretical basis for the design of chelators capable of crossing the blood–brain barrier and specifically correcting copper transporter dysregulation in neurons.

Furthermore, mitochondria are the key site where cuproptosis occurs; the development of mitochondria-targeted copper-chelating nanoparticles has been shown to effectively alleviate endothelial cell death and atherosclerosis induced by blood flow disturbances [171,172]. The historical failures of copper chelators in treating diseases including AD were precisely due to insufficient targeting, whereas FDX1 inhibitors or mitochondrial-targeted delivery represent the future direction. For example, a mitochondria-targeted copper-chelating nanoparticle delivery system can deplete copper ions to downregulate the expression of CD47 and PD-L1 in tumor cells, thereby overcoming innate and adaptive immune resistance. This demonstrates the immense therapeutic potential of targeting copper metabolism at the organelle level [173]. These novel strategies aim to achieve “precision chelation”—that is, eliminating pathogenic copper ions while preserving normal cellular copper homeostasis as much as possible—thereby opening new avenues for the treatment of neurodegenerative diseases.

Notably, not all modulatory strategies aim to eliminate copper; an alternative branch seeks to actively deliver copper so as to restore deficient functional pools. Cu-diacetyl-bis(N^4^-methylthiosemicarbazone) (Cu-ATSM) is a membrane-permeable, blood–brain-barrier-crossing Cu (II) carrier that selectively releases Cu^+^ in hypoxic tissues [174]. In *SOD1G93A* and *SOD1G37R* ALS mouse models, Cu-ATSM improved motor function and extended survival, at least in part by delivering copper to apo SOD1 and stabilizing the holo enzyme [26]; in Parkinson’s disease (PD) models it rescued the phenotype, and a phase I clinical trial in PD has yielded encouraging preliminary results [175]. However, although results from a recent study suggest that Cu-ATSM does not significantly alleviate neuronal pathology or astrocyte proliferation in patients with ALS [176], a subsequent commentary noted a significant reduction in spinal cord Iba1 microglial reactivity [177]. Thus, alongside “precision chelation,” the field is converging on a broader concept of precision copper modulation, in which the choice between chelating toxic copper or supplying functional copper must be tailored to the specific disease stage and the targeted copper pool—an integrative view that further underscores the need for organelle- and pathway-selective copper-targeting strategies.

### 5.3. Mitochondrial Protection and Metabolic Intervention

Mitochondrial dysfunction is a key upstream event in cuproptosis; therefore, strategies targeting the mitochondria represent an important avenue for intervening in this process. Mitochondria-targeted antioxidants, such as MitoQ, can accumulate specifically within mitochondria, effectively neutralizing the excessive ROS generated by copper overload, thereby alleviating mitochondrial oxidative stress [178].

Studies have shown that in a copper ion-induced neuronal cuproptosis model, the use of compounds with antioxidant activity, such as Icaritin, can significantly improve mitochondrial function. This includes restoring mitochondrial membrane potential, reducing ROS production, and regulating mitochondrial dynamics by promoting fusion and inhibiting fission, thereby protecting neurons from cuproptosis; Similarly, in a myocardial ischemia–reperfusion injury model, Phillygenin alleviated mitochondrial dysfunction in cardiomyocytes by inhibiting cuproptosis [179].

These findings suggest that directly protecting mitochondria from oxidative damage is an effective strategy for alleviating pathological processes associated with cuproptosis. In addition to antioxidant protection, enhancing the clearance mechanism for damaged mitochondria—namely, mitochondrial autophagy—is also a crucial approach to maintaining mitochondrial health. Studies have shown that regulating autophagy-related genes, such as *ATG5*, can influence the course of cuproptosis [180]. Therefore, the development of drugs capable of enhancing mitochondrial autophagy would help clear dysfunctional mitochondria, thereby alleviating mitochondrial stress upstream of cuproptosis at its source.

In addition to directly protecting mitochondria, enhancing cellular resistance to copper toxicity by modulating cellular metabolic pathways represents another highly promising intervention strategy.

The core mechanisms of cuproptosis involve lipid peroxidation pathways and the biosynthesis of iron–sulfur (Fe-S) clusters. Therefore, identifying small-molecule compounds that modulate these pathways could enhance cellular tolerance or directly inhibit cuproptosis. For example, studies have shown that glycosylated apigenin reduces copper influx by inhibiting low-density lipoprotein receptor-related protein 6, thereby suppressing cuproptosis in cardiomyocytes [181]. This suggests that targeting copper transporters is an effective approach for regulating intracellular copper homeostasis. On the other hand, key molecules in the lipoylation pathway can be directly targeted, such as the core targets of cuproptosis: multiple studies have confirmed that downregulating FDX1 expression via small-molecule inhibitors or genetic approaches effectively inhibits DLAT oligomerization, thereby blocking the cuproptosis pathway [178,182]; furthermore, modulating lipoylation-related enzymes such as LIAS is also a promising direction [179].

At the metabolic level, enhancing mitochondrial respiration through drugs (such as mitochondrial uncouplers) can increase the sensitivity of tumor cells to cuproptosis induced by copper ion carriers (such as elesclomol), offering a new combination strategy for cancer treatment [183].

In summary, the precise regulation of acylation metabolism, iron–sulfur cluster biosynthesis, or copper transport processes via small-molecule drugs can enhance cellular resistance to copper toxicity from multiple angles, offering new therapeutic approaches for treating cuproptosis-related diseases such as neurodegenerative and cardiovascular disorders.

## 6. Current Challenges and Future Research Directions

Despite the rapid accumulation of evidence linking cuproptosis to endothelial dysfunction and neurodegeneration, several fundamental controversies remain unresolved. Critically, these are not peripheral issues but strike at the very core of the pathway’s definition, its translational potential, and its distinction from other cell death modalities.

### 6.1. The Clinical Conundrum of Copper Chelation: Precision vs. Depletion

The most immediate translational hurdle is the historical failure of broad-spectrum copper chelators (e.g., penicillamine, clioquinol) in clinical trials for neurodegenerative diseases. While preclinically these agents reduce copper levels and attenuate pathology, human studies have yielded inconsistent results, with some even suggesting accelerated cognitive decline [184,185,186,187]. This paradox underscores a fundamental dilemma: systemic copper depletion lacks the spatial and subcellular precision required for therapeutic efficacy. The essential enzymatic functions of copper—within cytochrome c oxidase, SOD1, and dopamine β-hydroxylase—depend on a labile copper pool that chelators non-selectively deplete. Moreover, most conventional chelators poorly penetrate the BBB and fail to reach the mitochondria, the very organelle where cuproptosis is executed [70,170]. Recent data demonstrating that penicillamine protects against doxorubicin-induced cardiomyopathy by suppressing FDX1-mediated cuproptosis suggest that the beneficial effects may be highly context-dependent and require precise organelle targeting [169]. Thus, future success hinges not on stronger chelation but on smarter delivery: mitochondria-targeted nano-chelators or endothelium-adhesive carriers that discriminate between “toxic” and “functional” copper pools. Until such precision tools are developed, the chelation approach remains scientifically sound but clinically premature.

### 6.2. The Elusive Boundary Between Cuproptosis and Ferroptosis

Although cuproptosis is defined as a distinct cell death modality, its mechanistic overlap with ferroptosis raises a question: is cuproptosis genuinely autonomous, or is it a mitochondrial-restricted variant of ferroptosis triggered by specific redox metal imbalances? Both pathways converge on mitochondrial dysfunction, ROS production, and lipid peroxidation, albeit with different initiators (copper vs. iron) [100,188,189,190]. More compellingly, both pathways rely on the integrity of iron–sulfur (Fe-S) cluster biogenesis. Cuproptosis directly destabilizes Fe-S cluster proteins, while ferroptosis is modulated by Fe-S-dependent enzymes that regulate lipid peroxide repair [191,192,193]. In practice, distinguishing the two in complex tissues is methodologically challenging: the same cell may exhibit hallmarks of both pathways depending on the relative abundance of copper and iron, and the activation of one may deplete cellular antioxidants (e.g., GSH), sensitizing the cell to the other [39,42,100,189,190]. This cross-talk suggests that the binary classification of cell death modalities may be an oversimplification. Future studies should employ integrated multi-omics on single cells to dissect the sequential order of pathway activation rather than relying on a handful of markers. Therapeutically, simultaneous targeting of both copper and iron pathways might yield additive benefits, but this requires careful evaluation to avoid off-target systemic toxicity.

### 6.3. Cell-Type Specificity: Endothelium vs. Astrocytes vs. Neurons

A third controversy arises from striking cell-type differences in cuproptosis susceptibility. While neurons (especially dopaminergic and hippocampal neurons) appear highly susceptible to FDX1-dependent cuproptosis, astrocytes exhibit a markedly different response [8,194,195]. In vitro studies show that astrocytes are highly sensitive to copper toxicity, yet the mechanism does not fully align with classical cuproptosis—copper-induced astrocyte death is largely mediated by oxidative stress and does not require FDX1, suggesting a parallel, FDX1-independent copper toxicity pathway [196]. This is particularly relevant in the brain, where reactive astrocytes are prominent in AD and PD lesions. If astrocytes die via non-cuproptotic copper toxicity while neurons die via FDX1-dependent cuproptosis, then a drug that specifically inhibits FDX1 (e.g., small-molecule inhibitors) might protect neurons but leave astrocytes vulnerable, potentially creating an imbalance in glial–neuronal support. Conversely, a drug that broadly chelates copper might protect both but risks systemic depletion. This cell-type specificity is further compounded by microglia, which exhibit yet another sensitivity profile under inflammatory conditions. Therefore, therapeutic targeting of the cuproptosis pathway must account for this cellular heterogeneity [196]. The development of cell-type-specific delivery systems—e.g., peptide-conjugated nanoparticles that recognize endothelial adhesion molecules versus neuronal surface markers—will be essential to achieve the right therapeutic balance. Understanding these differential regulatory networks through single-cell RNA sequencing of diseased human tissues will be a prerequisite for rational drug design.

## 7. Discussion

The discovery of cuproptosis offers an unprecedented mechanistic lens through which to view the intersection of vascular pathology and neurodegeneration. This review has synthesized emerging evidence to advance a novel conceptual framework: the endothelial–neuronal cuproptosis axis. In this model, the cerebral endothelium is not merely a passive bystander but an active upstream hub. Endothelial cuproptosis dismantles BBB integrity, establishes a persistent SASP-driven inflammatory milieu, and, through the “copper overflow” mechanism, directly enriches the brain parenchyma with bioavailable copper ions. This copper-enriched microenvironment acts as a catalyst, synergizing with Aβ and α-synuclein pathologies to create a positive feedback loop that ultimately drives neuronal cuproptosis and cognitive decline.

Recognizing this vascular–neural continuum carries profound therapeutic implications. It suggests that intervening solely at the neuronal level—whether by clearing protein aggregates or delivering neurotrophins—may be insufficient if the vascular source of copper and inflammatory signals remains active. Conversely, preserving endothelial health through targeted anti-cuproptotic strategies might yield disease-modifying effects that are both upstream and broad-spectrum.

Looking ahead, the development of molecular imaging probes capable of distinguishing pathological copper accumulation (particularly within mitochondria and across the BBB) from physiological copper pools is urgently needed. Current PET tracers lack the specificity for lipoylated protein aggregates, and next-generation probes should be designed to detect FDX1 activity or DLAT oligomerization in vivo [42,197,198].

In addition, cell-type-specific and organelle-targeted delivery systems represent the holy grail of cuproptosis-based therapy. Endothelial-targeting peptides, exosome-engineered carriers, or nanotechnology-enabled mitochondrial delivery will be required to achieve “precision copper modulation”—eliminating the toxic copper pool without disturbing essential cuproenzyme functions [141,199,200].

Moreover, the field must resolve the temporal and contextual duality of cuproptosis. It remains unknown whether cuproptosis is primarily a pathogenic driver in early, preclinical stages (mild cognitive impairment) or an end-stage executioner in late disease. If the former, early biomarker-based screening and preventive intervention would be indicated; if the latter, symptomatic rescue strategies, rather than long-term modification, may be more relevant. Longitudinal studies in animal models and human cohorts with repeated biomarker sampling are essential to address this question.

In closing, cuproptosis stands at the crossroads of vascular biology, neuroimmunology, and mitochondrial metabolism. By embracing its systemic and cell-type complexity—rather than viewing it as a narrow, neuron-centric death pathway—we can transform this mechanistic insight into a new class of therapeutic strategies. The path from bench to bedside is challenging, but the potential to slow or prevent the most devastating neurodegenerative diseases of our age makes this pursuit not only scientifically compelling but urgently necessary.

## Figures and Tables

**Figure 1:**
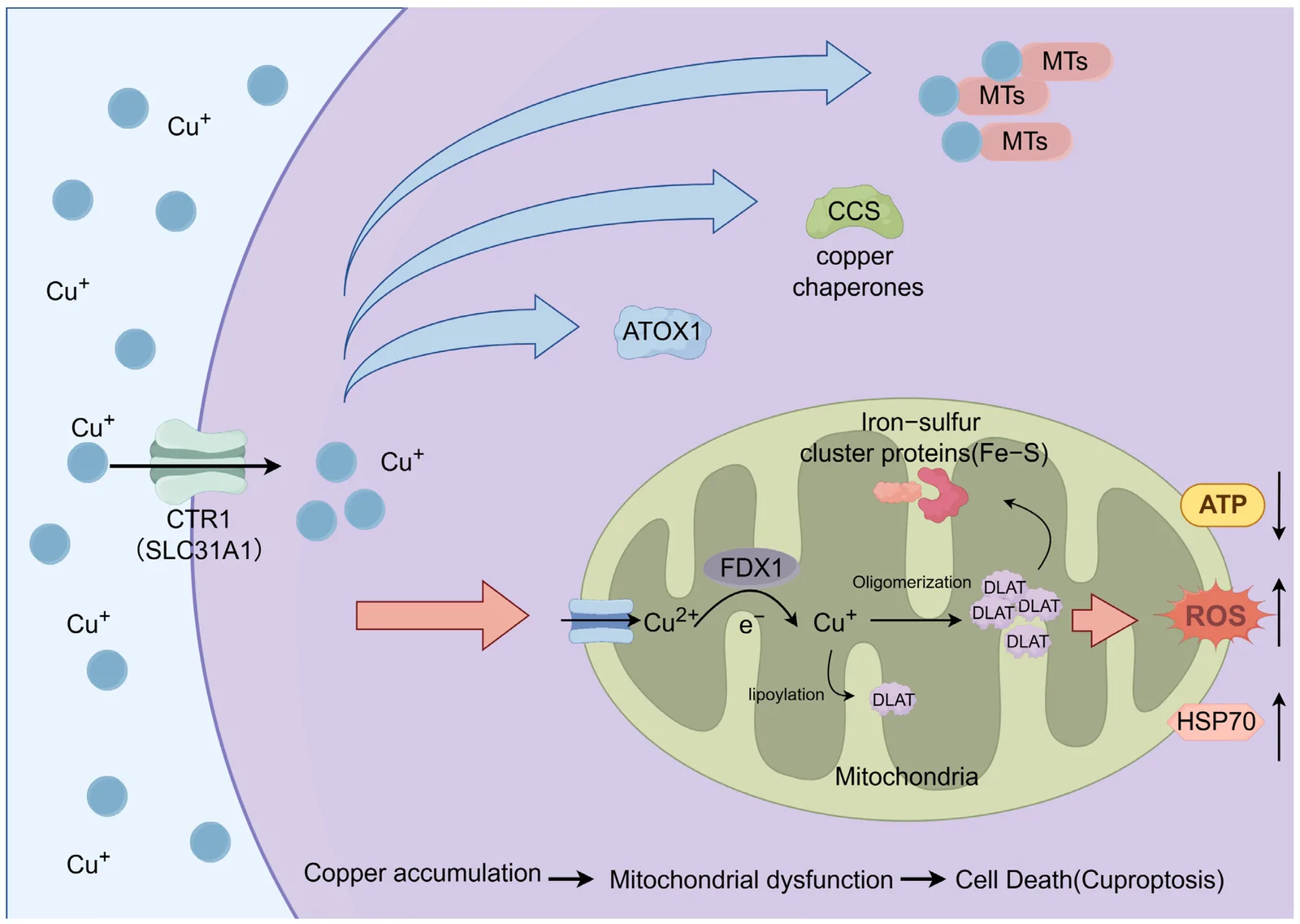
Core molecular events of cuproptosis in mitochondria. Excess copper ions (Cu^2+^) enter mitochondria and are reduced by FDX1. They bind to lipoylated TCA cycle enzymes, particularly dihydrolipoamide S-acetyltransferase (DLAT), inducing their aberrant oligomerization and aggregation. This simultaneously impairs iron–sulfur cluster biogenesis, causing loss of Fe-S proteins in mitochondrial respiratory complexes I–III. The combined effects disrupt TCA cycle flux and oxidative phosphorylation, leading to ATP depletion and cellular energy crisis. These hallmarks (protein aggregation, Fe-S loss, HSP70 upregulation) distinguish cuproptosis from other cell death modalities. The figure was created by Figdraw. Copyright Code: POPPW26656.

**Figure 2:**
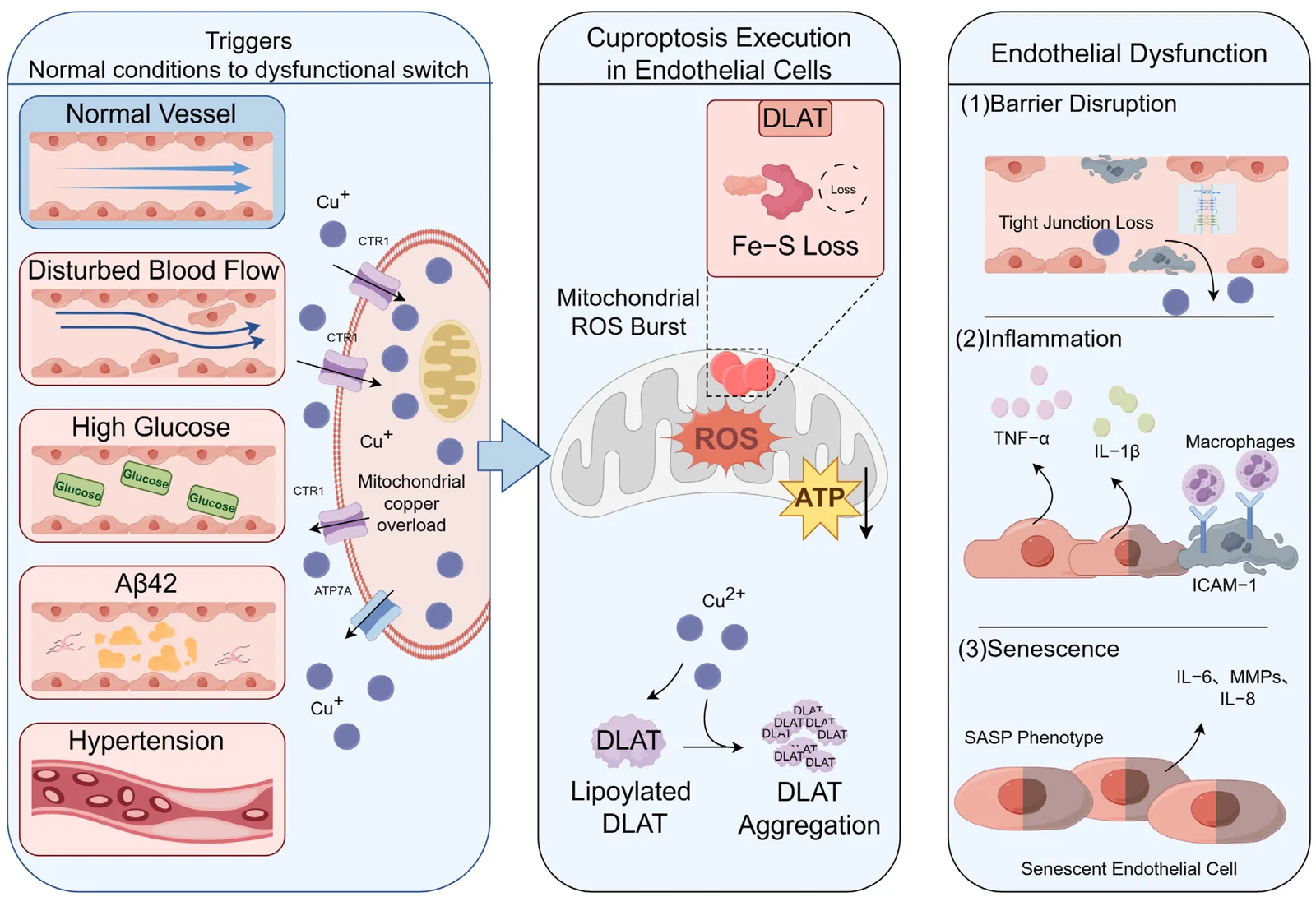
Copper-induced endothelial dysfunction: inflammation and senescence. In endothelial cells, mitochondrial copper overload triggers a ROS burst and mitochondrial dysfunction, driving the release of DAMPs and pro-inflammatory cytokines, as well as a senescence-associated secretory phenotype (SASP). Note: ROS production and SASP are hallmarks of copper-induced oxidative injury and toxicity; they are not part of the canonical cuproptosis pathway (which is ROS-independent and defined by FDX1-mediated lipoylated protein aggregation and Fe-S cluster loss). The figure illustrates the overall copper stress phenotype in the endothelium, encompassing both toxicity and, where applicable, cuproptotic components. The figure was created by Figdraw. Copyright Code: PISSTce999.

**Figure 3:**
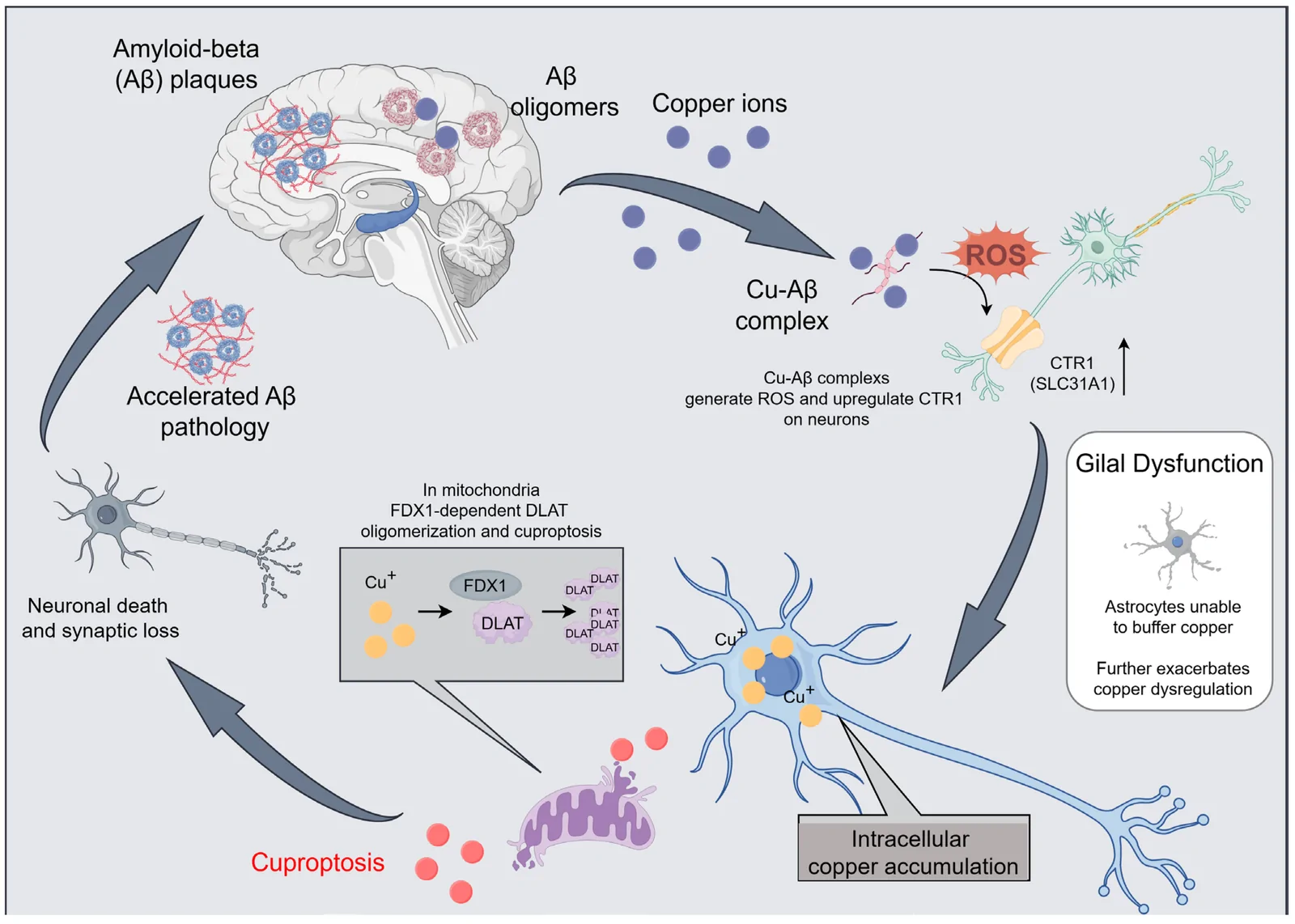
Cuproptosis in neuronal injury and neurodegenerative diseases. In Alzheimer’s disease, Aβ oligomers upregulate CTR1 and downregulate ATP7A, causing neuronal copper accumulation. Elevated Cu^+^ promotes ROS production and FDX1-dependent lipoylated protein aggregation, directly triggering cuproptosis. This pathway also impairs synaptic plasticity (via PSD-95 and SYP downregulation) and causes mitochondrial energy failure. In Parkinson’s disease and diabetic cognitive dysfunction, similar copper-mediated mitochondrial collapse and protein aggregation are observed. The figure illustrates how amyloid pathology and cuproptosis form a vicious cycle, accelerating neuronal loss and synaptic dysfunction in multiple neurodegenerative conditions. The figure was created by Figdraw. Copyright Code: OYAIO0f0e8.

**Figure 4:**
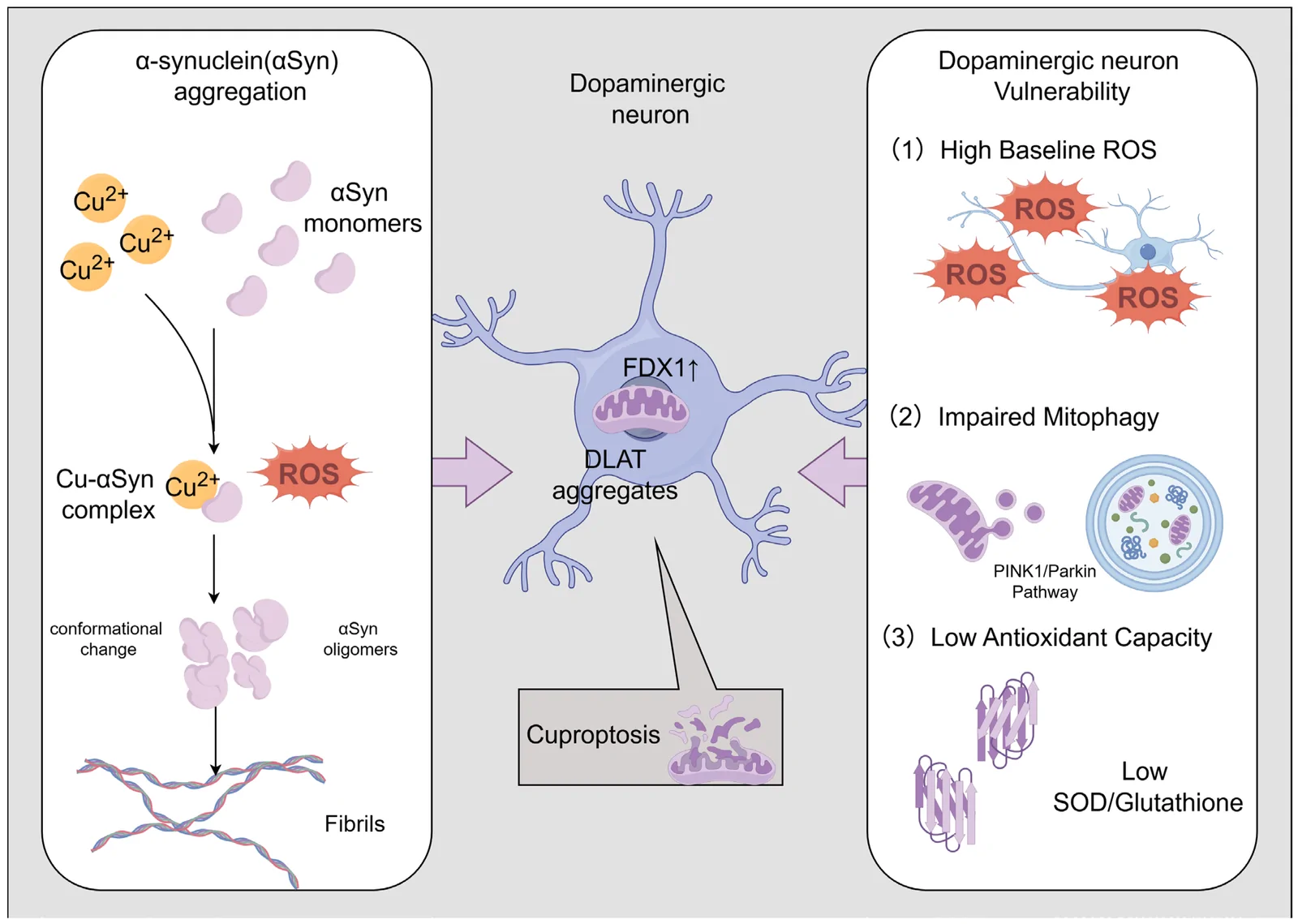
Vulnerability of dopaminergic neurons to cuproptosis in Parkinson’s disease. Dopaminergic neurons exhibit high metabolic activity and intrinsic oxidative stress. α-Synuclein (αSyn) binds copper ions via its N-terminal region, promoting conformational changes and toxic oligomer formation. The Cu–αSyn complex further disrupts copper homeostasis and mitochondrial function. In the presence of genetic defects (e.g., PINK1/Parkin mutations) or impaired mitophagy, damaged mitochondria accumulate, allowing copper to trigger cuproptosis through the FDX1/LIAS/DLAT axis. This figure summarizes the interplay between αSyn aggregation, copper dysregulation, mitochondrial dysfunction, and cuproptosis, explaining the selective degeneration of substantia nigra neurons in Parkinson’s disease. The figure was created by Figdraw. Copyright Code: PWPIU85885.

## Data Availability

No new data were created or analyzed in this study. Data sharing is not applicable to this article.
